# Quantitative ^1^H Nuclear Magnetic Resonance (qNMR) of Aromatic Amino Acids for Protein Quantification

**DOI:** 10.3390/mps6010011

**Published:** 2023-01-23

**Authors:** Teodor Tchipilov, Klas Meyer, Michael G. Weller

**Affiliations:** 1 Federal Institute for Materials Research and Testing (BAM), Division 1.5 Protein Analysis, Richard Willstätter-Strasse 11, 12489 Berlin, Germany; 2 Federal Institute for Materials Research and Testing (BAM), Division 1.4 Process Analytical Technology, Richard Willstätter-Strasse 11, 12489 Berlin, Germany

**Keywords:** amino acid analysis, AAA, protein hydrolysis, metrology, traceability, reference materials, internal standards, calibration

## Abstract

Hydrolysis of protein samples into amino acids facilitates the use of NMR spectroscopy for protein and peptide quantification. Different conditions have been tested for quantifying aromatic amino acids and proteins. The pH-dependent signal shifts in the aromatic region of amino acid samples were examined. A pH of 12 was found to minimize signal overlap of the four aromatic amino acids. Several aromatic compounds, such as terephthalic acid, sulfoisophthalic acid, and benzene tricarboxylic acid, were applied as internal standards. The quantification of amino acids from an amino acid standard was performed. Using the first two suggested internal standards, recovery was ~97% for histidine, phenylalanine, and tyrosine at a concentration of approximately 1 mM in solution. Acidic hydrolysis of a certified reference material (CRM) of bovine serum albumin (BSA) and subsequent quantification of Phe and Tyr yielded recoveries of 98% ± 2% and 88% ± 4%, respectively, at a protein concentration of 16 g/L or 250 µM.

## 1. Introduction

Protein analysis is still an increasingly active field of research. The tremendous variation in structure, as well as both physical and chemical properties, leads to many analytical challenges requiring that a variety of analytical techniques is employed. Protein quantification can be performed by elemental analysis, e.g., nitrogen by Dumas and Kjeldahl [1,2,3] or sulfur by ICP-MS [4,5]. Furthermore, various spectroscopic methods (UV absorbance and colorimetric reagents [6]), chromatography with UV or fluorescence detection [6,7,8,9], immunochemical methods for individual proteins [10], and many more can be used. The method of choice heavily depends on the application in question and the specific target analyte: A method appropriate for quantifying the protein content in food for nutritional purposes may be inappropriate for identifying and quantifying immunoglobulins in human serum.

Acidic hydrolysis into individual amino acids and subsequent analysis by high-performance liquid chromatography (total amino acid analysis, TAAA) is considered a gold standard for protein quantification of mixtures or pure compounds [6,11,12,13]. Both pre-column derivatization [6,14] and post-column derivatization [15,16] are useful for this purpose. Total hydrolysis of proteins into their amino acid constituents and subsequent quantification circumvent the impact of the protein’s structure on the analytical result. Hydrolysis conditions are typically 6 M hydrochloric acid at 107 °C for 24 h [6,11,12,13,14,15,16]. These conditions represent a compromise between protein hydrolysis efficiency and amino acid preservation. It often reduces the number of possible analytes to fewer than the common 22 proteinogenic amino acids. As amino acids differ in stability toward the hydrolysis conditions employed, the number of amino acids, which can be quantified, is often significantly lower. Some measures can be taken to alleviate the degradation of unstable amino acids during hydrolysis, and correction factors can sometimes be applied after analysis to account for analyte losses.

Recently, a modified variant of amino acid analysis was presented. Instead of trying to preserve and quantify as many amino acids as possible, hydrolysis and HPLC conditions are tailored to the aromatic amino acids L-tyrosine (Tyr) and L-phenylalanine (Phe), an approach which was termed aromatic amino acid analysis (AAAA). The technique was first used to quantify protein using separation by reversed-phase HPLC and subsequent UV detection [7]. Then, the method was modified using fluorescence detection, resulting in improved detection limits [8]. Furthermore, different stabilizers or protection agents were examined. Most recently, a variant using cysteine as a reducing agent and fluorescence detection was used on several analytes (pure and modified protein; protein in complex matrices) and compared favorably with other common protein quantification techniques [6]. The advantages of these AAAA techniques compared to TAAA are the lack of derivatization steps and the simpler, more time-efficient analysis. The hydrolysis time was successfully cut down to 1 h using ~8 M hydrobromic acid at 150 °C [6]. The separation of the analytes could be performed in less than 5 min per run. Tyr and Phe have been shown to be comparatively stable analytes under many conditions. However, in general, tyrosine is more sensitive than phenylalanine to unwanted reactions, such as nitration or halogenation [6]. Pritchard et al. counted Phe toward the pool of amino acids they considered stable for use in protein quantification via amino acid analysis, but not Tyr [17]. Nevertheless, when both are available at no additional expense, both should be evaluated.

Nuclear magnetic resonance spectroscopy (NMR) is a technique that relies on the presence of a nuclear spin that can be deflected using radio waves when placed in a strong magnetic field. Under the condition of full relaxation, the signal area under every signal is proportional to the number of nuclei responsible for that signal, making it a convenient tool for quantifying analytes (qNMR). One of the most important advantages of qNMR is compound-independent calibration. Hence, in contrast to mass spectrometry, for example, for which a different standard compound is usually required for each analyte, the choice of calibration standards for qNMR is much more flexible. If the standard is stable, well-behaved, and available in a pure form, and if the signals can be resolved, it may be used for quantification. However, the major limitation of qNMR is the poor sensitivity compared to most other detection methods.

NMR is a widespread and powerful tool in protein structure determination. Compared to diffractometric X-ray techniques, the sample does not need to be crystallized. Spectra can be collected in a variety of different liquid media that may have more practical relevance than the entity present in a highly ordered crystal. Proteins are large biomolecules with molar masses of many kilodaltons. Their irregular shape, limited symmetry, and large size are of significant importance when measuring their NMR spectra. If several units of the same amino acid are present in the protein, they are unlikely to be chemically and magnetically equivalent, meaning that they display differing resonance frequencies for the nuclei being observed. One-dimensional ^1^H-NMR spectra are likely to have poor signal-to-noise ratios and highly overlapping signals, making further analysis challenging. Carbon NMR spans a wider frequency band than ^1^H-NMR, leading to less convolution of individual signals, but the low natural abundance of ^13^C-carbon means that signal-to-noise ratios are substantially worse. If budget and context allow, this may be counteracted in part by using ^13^C- and ^15^N-enriched amino acids for protein synthesis [18,19]. This technique, along with others such as polarization transfer and multidimensional spectra, is commonly utilized for NMR spectroscopy for the purposes of structural elucidation of proteins [19,20,21]. The latter are not commonly used in qNMR as they break the universal proportionality of the signal area with analyte concentration by introducing a group- and signal-dependent bias to the measurement. qNMR measurements, for this reason, are commonly the simplest variant of the NMR experiment. A single pulse is followed by reading the resulting free induction decay.

Due to the molecular properties of the intact analytes, the qNMR of protein is challenging. Efforts to quantify peptides of a limited length and individual amino acids have been made [22,23,24,25,26]. Digestion of intact protein into individual amino acids provides an avenue for qNMR analysis; cleaving a protein into individual amino acids enhances the signal-to-noise ratio by the respective number of the respective amino acid by making these amino acids chemically equivalent. However, this process may also introduce some bias. The harsh hydrolytic conditions of such digestion may degrade some amino acids, at least partially, while the protein chain might not have been cleaved completely.

The focus on aromatic amino acids, especially on tyrosine and phenylalanine, for this work has several advantages. Their signals are found in a region of the spectrum that is typically not busy. Byproducts or impurities are unlikely to interfere, which substantially simplifies the analysis of the measured one-dimensional spectra. Additionally, tyrosine and particularly phenylalanine [6] have been shown to be stable analytes under the chosen hydrolytic conditions and are suitable for the quantification of known proteins. If the type of protein and its amino acid composition is known, the protein quantity is readily available by measuring one or several of its amino acids and multiplying the molarity or mass appropriately. The use of volatile mineral acids such as hydrochloric and hydrobromic acids allows the user to remove the bulk of the reagent to be more flexible in the final solvent.

The present work explores a combination of AAAA and qNMR with the goal of combining the robustness of the aromatic amino acids tyrosine and phenylalanine as analytes and the traceability of qNMR for use in, for instance, quantification of protein standards, in addition to the production of certified reference materials (CRM). Signals of the aromatic residues of the common aromatic amino acids histidine, phenylalanine, tyrosine, and tryptophan were measured in samples containing individual amino acids, and their chemical shifts were observed after the variation of pH. In the second step, several aromatic compounds were examined as internal standards at different pH values. In the third step, a mixture of aromatic amino acids was quantified with an internal standard to determine the respective recoveries. Lastly, a certified reference material of bovine serum albumin (BSA) was hydrolyzed, quantified, and compared with the certified values.

## 2. Materials and Methods

### 2.1. Materials

Mono-, di-, and trisodium phosphates (di-, di-, and dodecahydrate; p.A., BioChemica and p.A.) were purchased from AppliChem GmbH (Darmstadt, Germany). Phosphoric acid (85%), concentrated hydrochloric acid (37%), deuterium oxide (99% and 99.99% D), amino acids (synthesis grade), and amino acid standards (analytical standard, 2.5 mM, in 0.1 M HCl) were obtained from Sigma-Aldrich (St. Louis, MO, USA). The compounds chosen were obtained from the following sources: 1,3,5-benzene-tricarboxylic acid (trimesic acid, BTCA, synthesis grade), terephthalic acid (TPA, 98%), disodium 1,3-benzenedisulfonate (BDSA, for synthesis), and 1,5-naphthalenedisulfonic acid (NDSA, 97%) from Sigma-Aldrich (St. Louis, MO, USA), along with potassium 4-sulfobenzoate and potassium 5-sulfoisophthalate (SBA and SIPA, both 95%) from Alfa Aesar GmbH (Karlsruhe, Germany). It should be stressed that, in most cases, the commercial purity of these compounds is insufficient to be used as an internal standard without further purification and characterization. They were used mainly for screening purposes here. As a certified reference material, bovine serum albumin (BSA), NIST SRM 927e was used (7% in water).

### 2.2. Preparation of Deuterated Phosphate Buffers

First, 0.1 M solutions of phosphoric acid, mono-, di- and trisodium phosphates were prepared. To obtain buffers at approximately pH 2, 7, and 12, equal volumes of phosphoric acid and monosodium phosphate, mono- and disodium phosphate, and di- and trisodium phosphate solutions were mixed. Then, 1 mL aliquots of the resulting buffers were reduced to dryness over 20 h at 40 °C and 100 mbar of pressure. In order to achieve a greater degree of deuteration, the buffers were dissolved in deuterium oxide (99% D) and reduced to dryness again, after which they were stored in a desiccator. Lastly, the buffers were dissolved in deuterium oxide (99.99% D) immediately before use.

### 2.3. Hydrolysis of Bovine Serum Albumin (BSA)

Water was added to a sample-dependent amount of BSA solution (NIST 927e, 7% by weight) to achieve a total volume of 1 mL. Then, 1 mL of concentrated hydrochloric acid (37% by weight, ca. 12 M) was added to this sample, and the mix was transferred into a Schlenk tube. In order to remove oxygen, a vacuum was applied and flushed with argon. This step was repeated thrice. After that, the tube was closed in an evacuated state and heated to 107 °C for 24 h in an aluminum heating block. Upon completion, 1 mL of hydrolysate was reduced to dryness over 20 h at 40 °C and 100 mbar of pressure. Lastly, the dry sample was reconstituted using a 1 mL aliquot of deuterated phosphate buffer containing 3.3 mM of BTCA as the internal standard.

### 2.4. Preparation of Amino Acid and Standard Candidate Solutions

For the signal shift assessments, approximately 1 mg of the amino acids or standard compounds were dissolved in 0.75 mL of deuterated phosphate buffer, pH 12.

### 2.5. Preparation of TPA, SIPA, and Amino Acid Standard Solutions for Recovery Measurements

First, 2 mM TPA and SIPA solutions were prepared by weighing 35.05 and 53.60 mg of TPA and SIPA into volumetric flasks and dissolving them in water. TPA required the addition of small quantities of sodium hydroxide solution to dissolve fully. After this, both solutions were filled to 100 mL. Then, 25 to 400 µL of amino acid standard (2.5 mM in 0.1 M hydrochloric acid) and 975 to 600 µL of water were mixed with 175 µL of TPA or SIPA standard. The samples were then reduced to dryness (40 °C, 900 mbar of vacuum, 20 h) and reconstituted in 1 mL of deuterated phosphate buffer (pH 12, 0.1 M).

### 2.6. Acquisition and Evaluation of NMR Spectra

A Varian VNMRS 500 (spectrometer frequency 499.89 MHz) with a 5 mm OneNMR probe was used to acquire all spectra. The number of scans was eight for qualitative and 64 for quantitative measurements unless specified otherwise. In an inversion recovery experiment (data not shown), T1 for all aromatic protons was found to be below 5 s; thus, relaxation delays between pulses were chosen to be 5 s for qualitative and 30 s for quantitative measurements. Chemical shifts were referenced to the spectrometer frequency instead of a standard as the experiments focus on signal separation and shifts in the same sample. Fourier transformations and phase corrections were performed with SpinWorks 4.2.9, after which the data were exported into Origin 2019 (Version 9.6.0) for integration and visualization and Excel for general calculations.

## 3. Results and Discussion

### 3.1. Proteins as an Analyte in (q)NMR

**Figure 1:**
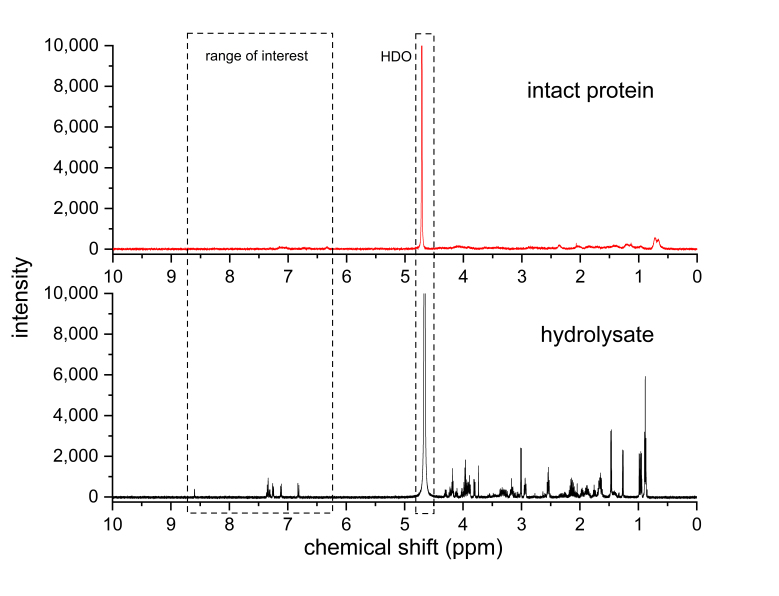
^1^H-NMR spectra of intact (red trace, top) and hydrolyzed protein (black trace, bottom). Approximately 1 mg/mL of protein (BSA) or protein hydrolysate in D_2_O, 500 MHz spectrometer frequency, 16 scans, 5 s relaxation delay, 30° observation pulse, and 0.5 Hz Lorentzian apodization. The large signal at around 4.7 ppm is that of residual water (as protium deuterium oxide, HDO).

As outlined before, intact protein presents severe challenges for qNMR analysis that may be alleviated through digestion. BSA, a common model protein that is readily available even as certified reference material (CRM), has a molecular mass of around 66 kDa. Figure 1 shows a comparison of spectra of intact and acid-hydrolyzed BSA of the same concentration. In the spectrum of the intact protein, essentially, no clear signals are visible. In contrast, the signals of the hydrolysate are narrow, and their signal-to-noise ratios are much higher. The hydrolysate spectrum is busy in the chemical shift range below the water signal, but the aromatic region is very promising. Notably, Phe and Tyr signals are present after hydrolysis under these conditions, and the signals for these aromatic protons are baseline-separated at acidic pH, allowing for easy integration and quantification if desired.

### 3.2. The pH Dependency of the Chemical Shifts

The pH-dependent protonation of functional groups influences the electron density and, hence, the chemical shifts. This has been shown in the literature, e.g., for asparagine and the indole proton of histidine [19,27]. The magnitude of the influence of pH depends highly on the chemical structure of the analyte. A phenyl residue is less prone to this effect than the residues of the other aromatic amino acids. Therefore, pH variation can be used to optimize the separation of signals of interest.

Phosphoric acid is a convenient mineral acid to use for testing since it is tribasic with pKa values of 2.2, 7.2, and 12.4 [28], and buffers of it are common, easy to prepare, and can be restored when dried and redissolved with deuterium oxide. Phosphate buffers with a pH (2.0, 7.0, and 12.0) near their pKa values were prepared, aliquoted, reduced to dryness, and reconstituted with deuterium oxide shortly before use. The reconstituted buffer was then used to dissolve the amino acids or protein hydrolysates for NMR measurements.

**Figure 2:**
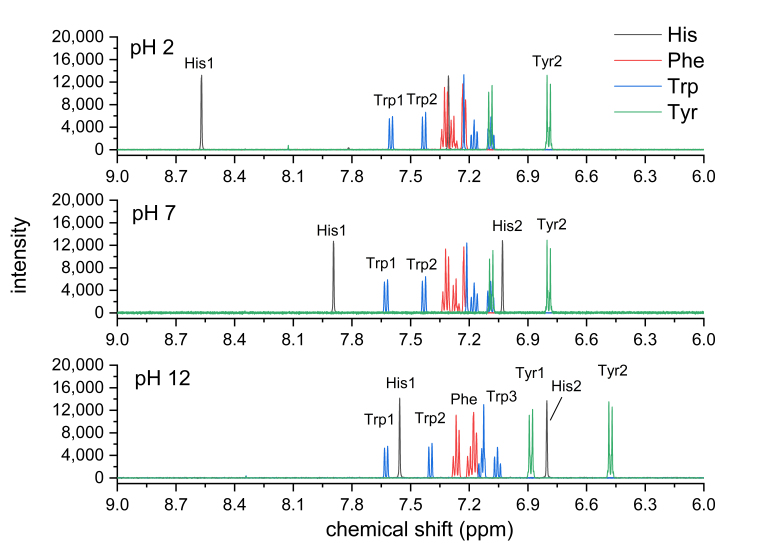
The pH-dependent signal shift in the aromatic region at pH 2, 7, and 12. Approximately. 1 mg/mL of each amino acid in deuterated 100 mM phosphate buffer 500 MHz spectrometer frequency, 16 scans, 5 s relaxation delay, 30° observation pulse, and no apodization. Signals observed in the pH 2 spectrum at ~7.8, and ~8.1 ppm may have been caused by non-identified impurities.

**Figure 3:**
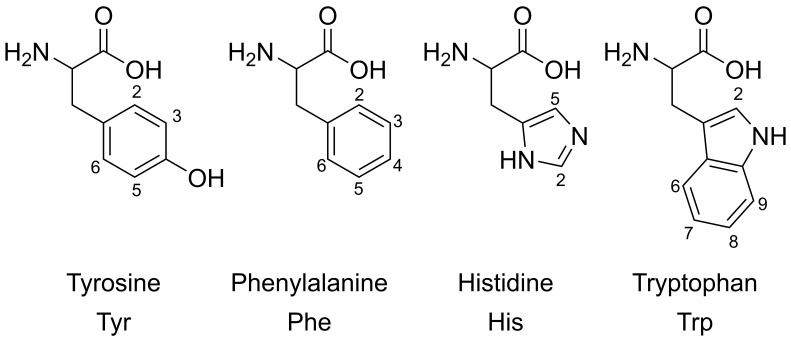
Structures and annotated aromatic protons of relevant amino acids (chirality and zwitterionic forms are not shown).

The overlay of individual amino acid spectra is shown in Figure 2. Measurements at pH 2, 7, and 12 showed significant chemical shift changes for the aromatic proton signals of His, Tyr, and Trp. As expected, the aromatic residue of phenylalanine (red line) was largely unaffected by pH. At pH 2 and 7, some overlapping signals were observed, while, at pH 12, baseline separation for almost all signals and signal groups in the aromatic region was achieved. Figure 3 and Table 1 show the structures of the four aromatic amino acids and their chemical shifts of the aromatic proton signals for all three pH values. Due to the better signal separation, we chose pH 12 for all subsequent measurements.

### 3.3. Selection of Potential Compounds as Internal Standards for qNMR of Aromatic Amino Acids

Commercial CRMs and standards for aqueous qNMR are somewhat limited in their availability. Ideally, for use in aromatic amino acid quantification, its signal should be found in the aromatic proton region of the spectrum. Promising candidates are organic acids such as formic, phthalic, and maleic acids, as well as their salts. According to the documentation of a reagent supplier [29], these compounds show chemical shifts of 8.4, 7.5, and 6.3 ppm, respectively. This places phthalic acid’s signal potentially in the cluster of amino acid signals. While their solubility in acidic solutions may be limited, a pH of 12 would allow their use. Furthermore, dimethyl terephthalate is commercially available as a CRM and may potentially be used after in situ hydrolysis with the protein. In addition to terephthalic acid, several carboxyl- and sulfonyl-substituted aromatic compounds were evaluated for their use as standards in this application.

**Figure 4:**
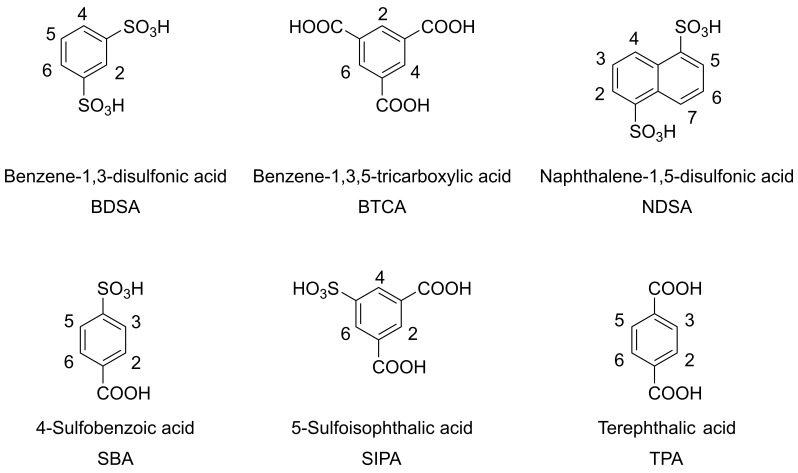
Structures of candidate compounds potentially useful as internal standards: benzene-1,3-disulfonic acid (BDSA), benzene-1,3,5-tricarboxylic acid (BTCA), naphthalene-1,5-disulfonic acid (NDSA), 4-sulfobenzoic acid (SBA), 5-sulfoisophthalic acid (SIPA), and terephthalic acid (TPA).

**Figure 5:**
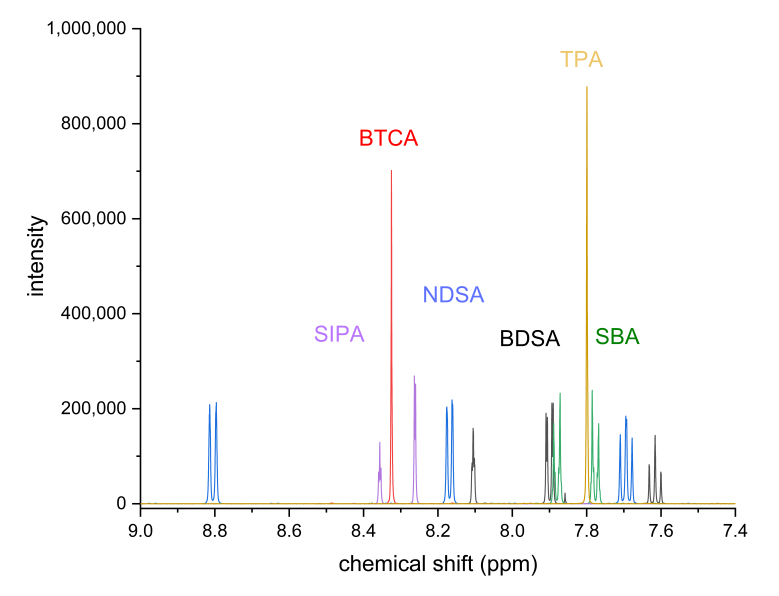
Aromatic proton signals for the selected standard candidates at pH 12 (for abbreviations, see Figure 4). BDSA and NDSA signals may overlap with amino acid signals that reach up to 7.65 ppm and, therefore, are less useful for this application. The concentrations were around 1 g/L in deuterated phosphate buffer (100 mM, pH 12); 500 MHz spectrometer frequency, 16 scans, 5 s relaxation delay, 30° observation pulse, and no apodization.

Figure 4 and Figure 5 show structures and ^1^H-NMR spectra of terephthalic acid (TPA), 4-sulfobenzoic acid (SBA), 5-sulfoisophthalic acid (SIPA), benzene-1,3-disulfonic acid (BDSA), naphthalene-1,5-disulfonic acid (NDSA), and benzene-1,3,5-tricarboxylic acid (BTCA). These compounds are readily available, even though further purification for use as standard would be required; their substitutions with electron-withdrawing functional groups shift the signals to a higher field, outside of the region of the spectrum in which the amino acid signals are found, and, in the case of sulfonic acids, their solubility should be no concern regardless of pH. As shown in the spectra, NDSA and BDSA signals were not quite downfield shifted enough, risking overlaps with amino acid signals. SBA, BDSA, and NDSA, due to lack of symmetry, show multiple signals. As a result, BTCA, TPA, and SIPA show the most promise. BTCA offers an even higher chemical shift than TPA if required but being trisubstituted results in less signal area per amount of standard used. SIPA offers solubility at low pH and may be added before acidic hydrolysis for the user to account for losses during sample handling. However, its stability during this process still needs to be assessed. Because the high solubility offers a tangible benefit compared to TPA, it was also used for further measurements. This selection of standards is by no means exhaustive, as qNMR spectroscopy allows tremendous flexibility in the choice of calibration standards.

### 3.4. Quantification of Aromatic Amino Acids from a Commercial Standard Solution

Since protein quantification via its amino acid constituents is a proven technique, tailored reagents are commercially available. Therefore, we used such standard with most proteinogenic amino acids at a 2.5 mM concentration in 0.1 M hydrochloric acid to assess the concentration range and recovery of the qNMR technique discussed in this study, along with TPA and SIPA internal standards for the application with protein and amino acid samples.

**Figure 6:**
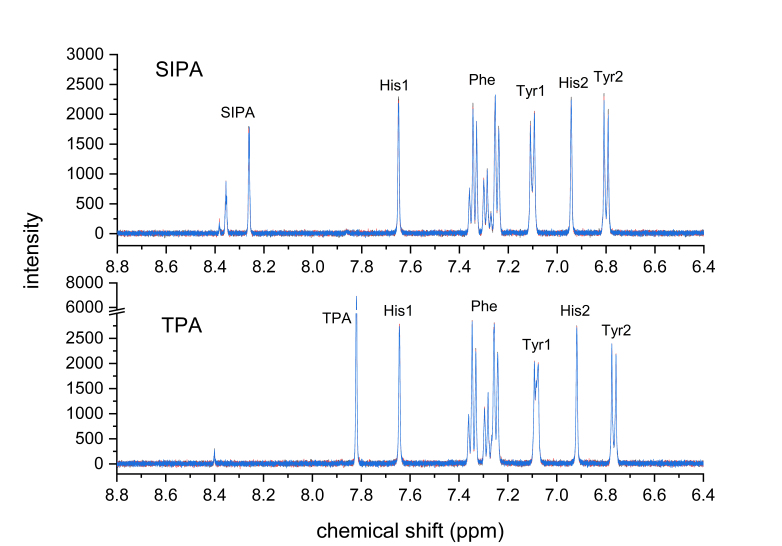
Aromatic proton regions of technical ^1^H-qNMR spectra triplicates (overlays) of amino acid standard samples at 1 mM in deuterated phosphate buffer (100 mM, pH 12); 500 MHz spectrometer frequency, ns = 128, d1 = 30 s, 30° observation pulse, and no apodization.

Figure 6 shows the aromatic region in ^1^H-qNMR spectra of the highest-concentration samples measured in this series, corresponding to 1 mM of His, Phe, and Tyr, along with 0.35 mM of the internal standards. This concentration of amino acids would correspond to approximately 0.03 to 0.05 mM or 2 to 3 mg/L of BSA in the sample. However, because the sample is hydrolyzed and reduced to dryness, the user has some flexibility in choosing how much sample is used. Recoveries for the SIPA concentration series can be found in Table 2. As expected, the values deviated more as the analyte concentration, and signal-to-noise ratio decreased. The TPA series mirrored these values (Table 3).

The data shown in Table 2 and Table 3 were derived from integrating the signals of the chosen internal standard and all amino acids observed. Adjacent signals of the same compound were combined and integrated together. For SIPA, the triplet and singlet signals were combined and integrated together. The amino acid concentration in the NMR sample was then given as the ratio of signal areas of the amino acid and the internal standard signals, weighted by the number of protons included in those signals, and multiplied by the internal standard concentration. Errors were estimated as being the sum of the relative standard deviation of both the internal standard and the amino acid signal areas multiplied by two.

For a still unknown reason, the signal Tyr2 exhibits lower-than-expected recoveries even at high concentrations. Regardless, tyrosine could still be quantified using the redundant signal Tyr1. Both potential internal standards, TPA and SIPA, are deemed suitable for this analysis.

### 3.5. Quantification of a Protein (BSA) by the Analysis of Aromatic Amino Acids

The method’s goal was its application to protein samples; hence, acidic hydrolysis and subsequent quantification of an example protein were performed. Bovine serum albumin (BSA) is widely used for these purposes and is available as certified reference material. For this study, NIST SRM 927e, a 7% solution of BSA, was hydrolyzed and analyzed using BTCA as an internal standard at a concentration of 3.3 mM. Hydrolysis was performed using 6 M hydrochloric acid and heating to 107 °C over 24 h as opposed to using concentrated hydrobromic acid at 150 °C over 1 h, as demonstrated recently due to this being the more established technique [6].

**Figure 7:**
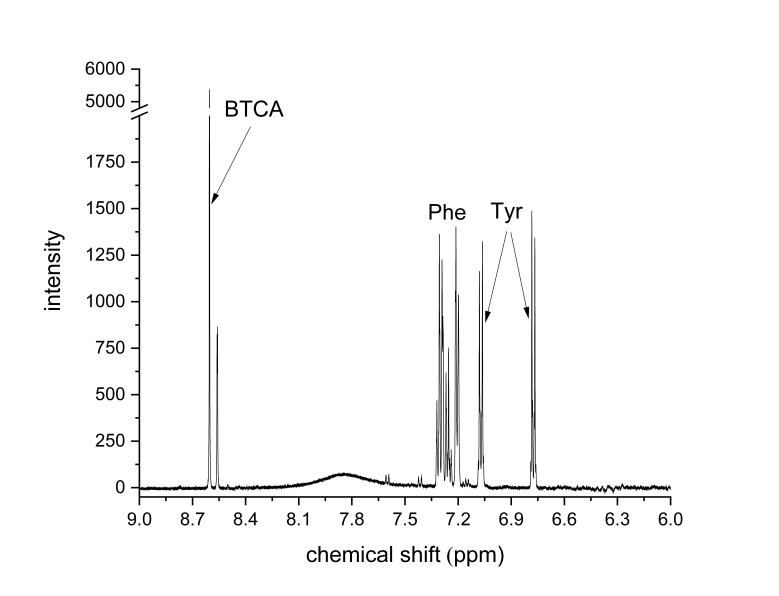
Aromatic region of ^1^H-qNMR spectrum of hydrolyzed BSA, 0.25 mM, using 3.3 mM of BTCA as internal standard; pH 12; ns = 128, d1 = 30 s, 30° observation pulse, and no apodization.

Figure 7 shows the aromatic region of the ^1^H-qNMR spectrum. Table 4 shows the measured parameters for two amino acids, Phe and Tyr.

Signals for the three compounds were found at 8.6 (BTCA), around 7.3 (Phe), and at both 7.1 and 6.8 ppm (Tyr). Furthermore, a broad signal of unknown origin was found at 7.85 ppm. Measured recoveries for both amino acids were 96% ± 2% for Phe and 86% ± 5% for Tyr.

The tyrosine recovery was noticeably worse than that of phenylalanine, which was nearly quantitative. Tyrosine being less stable than phenylalanine has already been demonstrated [6]. The qNMR step is independent of the specific hydrolysis protocol used for the sample. Therefore, the shortened hydrolysis methodology published recently may be used [6].

Technical replicates of previous samples indicated a relative standard deviation of approximately 0.5 %. Three independent samples deviated in the order of 2% (Phe) and 5% (Tyr), respectively. Given the relatively complex workflow, most of the variance may have arisen from the sample preparation.

Approaches to improve the methodology presented here may be straightforward. Both the hydrolysis and the NMR measurement could be improved further. Depending on which amino acids are intended to be used for quantitation, the hydrolysis and sample preparation steps need to be adopted. Basic hydrolysis may allow the quantification of tryptophan [8]. In addition, if more of the sample volume is reduced to dryness, higher sensitivity should be achieved.

#### Outlook and Future Applications

Further improvement of the sensitivity of the NMR spectroscopic method is possible by either increasing measurement time or changing to different hardware. The first approach is following a square-root law, which quickly leads to non-appropriate time requirements. Therefore, changing to hardware with a higher performance would be preferable. An increase in the magnetic field strength by a factor of two would theoretically lead to an improvement of the signal-to-noise (SNR) factor of up to 2.8. A powerful way would be the use of NMR cryoprobes, reducing the influence of thermal noise in the primary RF detection circuit. With this type of equipment, an SNR increase by a factor of 2–4 is possible depending on the sample properties [30]. However, the high operation and maintenance costs of the cooling systems need to be considered.

In addition, spectral modeling approaches such as global spectral deconvolution or indirect hard modeling can be options to improve signal area determination compared to numerical integration, especially in automated setups and limited SNR [31,32]. Lastly, in applications aimed at the determination of free amino acids, hydrolysis would not be required at all. Phenylketonuria, for instance, is characterized by the ratio of the amino acids phenylalanine to tyrosine and an elevated concentration of phenylalanine in the serum around 1 mmol/L [33,34]. This concentration is well within the concentration range covered by this work. Other samples in which free amino acid concentrations are relevant could be honey, yeast extract, meat broth, soy sauce, and bacterial culture media.

## 4. Conclusions

Acidic hydrolysis of protein into amino acids is a well-established technique for quantitative protein analysis. Today, qNMR of small molecules is a relatively common technique for purity determinations and metrological applications. Combining the two techniques allows the quantification of proteins via their amino acid constitution, leading to a method potentially traceable to the International System of Units (SI). This approach is applicable to all proteins and peptides that contain at least one aromatic amino acid in their sequence. As a result, the method might be used for nearly all proteins, many peptides, and samples containing free (aromatic) amino acids.

The standard method for acidic hydrolysis using 6 M hydrochloric acid at 107 °C over 24 h is perfectly suitable for this technique. However, other variants, such as higher temperatures, e.g., at 150 °C or the use of microwave digestions, would likely be applicable. The pH for the NMR measurement can be flexibly selected according to individual requirements; a phosphate buffer of pH 12 was chosen in this work. Some new compounds potentially useful as internal standards have been tested. Sulfonic acids show advantages in solubility, and terephthalic acid, particularly its potassium salt, is available in higher purity.

## Figures and Tables

**Table 1:** Chemical shifts and other parameters of aromatic proton signals.

Signal	Proton Assignment	Multiplicity	δ (pH 2) (ppm)	δ (pH 7) (ppm)	δ (pH 12) (ppm)
Trp1	7	D	7.59	7.62	7.62
His1	2	S	8.56	7.89	7.55
Trp2	6	D	7.43	7.43	7.39
Phe	2, 3, 4, 5, 6	M	7.27	7.26	7.26
Trp3	2, 8, 9	M	7.13	7.13	7.09
Tyr1	2, 6	D	7.09	7.09	6.88
His2	5	S	7.30	7.03	6.80
Tyr2	3, 5	D	6.79	6.79	6.47

**Table 2:** Recoveries (%) of the amino acid series using SIPA to quantify each signal.

Sample (µM)	His1 (%)	Phe (%)	Tyr1 (%)	His2 (%)	Tyr2 (%)
1000	97	97	97	100	82
500	104	100	100	102	66
250	105	105	103	108	72
125	112	113	114	118	104

**Table 3:** Recoveries (%) of the amino acid series using TPA to quantify each signal.

Sample (µM)	His1 (%)	Phe (%)	Tyr1 (%)	His2 (%)	Tyr2 (%)
1000	95	97	96	98	68
500	95	96	95	99	68
250	99	101	100	101	98
125	128	113	113	119	114

**Table 4:** Signal areas, concentrations, and resulting recoveries for the hydrolysis and subsequent analysis of BSA hydrolysate; Concentrations of the reference material are certified with an expanded uncertainty of approximately 2 %.

Signal	Signal Area (a.u.)	RSD (%)	Expected (mM)	Measured (mM)	Recovery (%)
BTCA	14.1	0.5	3.3	-	-
Phe (5 protons)	47.0	2	6.8	6.5	96 ± 2
Tyr1 (2 protons)	12.7	5	5.1	4.4	86 ± 5

## Data Availability

Not applicable.
